# Identifying the Uncertainty in Physician Practice Location through Spatial Analytics and Text Mining

**DOI:** 10.3390/ijerph13090930

**Published:** 2016-09-21

**Authors:** Xuan Shi, Bowei Xue, Imam M. Xierali

**Affiliations:** 1 Department of Geoscience, University of Arkansas, Fayetteville, AR 72701, USA; boweixue@email.uark.edu; 2 Association of American Medical Colleges, Washington, DC 20001, USA; ixierali@aamc.org

**Keywords:** spatial uncertainty, physician distribution, spatial analytics, text mining, visual examination

## Abstract

In response to the widespread concern about the adequacy, distribution, and disparity of access to a health care workforce, the correct identification of physicians’ practice locations is critical to access public health services. In prior literature, little effort has been made to detect and resolve the uncertainty about whether the address provided by a physician in the survey is a practice address or a home address. This paper introduces how to identify the uncertainty in a physician’s practice location through spatial analytics, text mining, and visual examination. While land use and zoning code, embedded within the parcel datasets, help to differentiate resident areas from other types, spatial analytics may have certain limitations in matching and comparing physician and parcel datasets with different uncertainty issues, which may lead to unforeseen results. Handling and matching the string components between physicians’ addresses and the addresses of the parcels could identify the spatial uncertainty and instability to derive a more reasonable relationship between different datasets. Visual analytics and examination further help to clarify the undetectable patterns. This research will have a broader impact over federal and state initiatives and policies to address both insufficiency and maldistribution of a health care workforce to improve the accessibility to public health services.

## 1. Introduction

Understanding the spatial distribution of physicians has a significant impact on both policy-making and accessibility studies to enable the patients to find reliable public health services. Physician shortages due to physician workforce maldistribution is a significant feature and concern of the US health care system [1,2]. Consequently, health disparity issues have been argued, particularly when the shortage of physicians would be obvious and serious in certain areas. Significant geographic maldistribution of a physician workforce has been widely documented across and within the rural/urban stratum [3,4,5,6]. Federal incentive programs, such as Health Professional Shortage Areas (HPSAs) and Medically Underserved Areas and Populations (MUAPs), were established to mitigate the effect of and to correct for the spatial maldistribution of health care resources [4,7]. For the general public, access to medical or health service providers has been critical to the patients, especially when the coverage expansion in the Patient Protection and Affordable Care Act [8] is extended to millions of Americans.

The spatial distribution of physicians can be derived from a variety of data sources, such as the Physician Masterfile maintained by the American Medical Association (AMA), the National Provider Identifier (NPI) database as provided by the Centers for Medicare and Medicaid Services (CMS), and state medical licensure data. Such data sources contain addresses of the physicians who responded to the surveys conducted by different professional associations and administration agencies. Traditionally, Geographic Information System (GIS) software is used to implement the geocoding function that converts the addresses of physicians or patients into geographic coordinates and displays the location of physicians or patients on the map along with other data layers to visualize the spatial distribution of physicians or patients. For this reason, most of the prior works discussed the potential inaccuracy problems in the address or the spatial errors in the geocoding results [9,10,11,12,13,14,15,16,17].

In understanding the spatial distribution of physicians, however, it is of particular significance and interest to clarify whether the address provided by the physician is the home address or the practice address for the clinics or relevant healthcare services. Since the practice address is the key to understand relevant issues of maldistribution, accessibility and disparity, it is not meaningful if the home address is blended within the varieties of data sources. Differentiating the home addresses from practice addresses is beyond the capability of the geocoding function. Auxiliary data and solutions have to be utilized to resolve such an uncertainty problem.

At the state or county level, parcel data have been created and updated for tax commissioners to evaluate and determine the value of the property as the key source of the revenue income [18,19]. For this reason, parcel data have to be accurate and up-to-date and contain the zoning code that specifies the land use types, from which it can be clarified whether the parcel is utilized as a single family or multi-family resident community, for commercial or industrial development, or as tax exempted areas including governmental properties, churches, hospitals, non-profit organizations, and so on. Since parcel data contain the address information of the property, consequently, it can be a promising resource to clarify whether the address provided by the physician is the home address or not, when the physician’s address can be matched to the parcel dataset.

Matching and comparing the address information in the physician datasets to the addresses in the parcel datasets would have generated a variety of unforeseen challenges or problems. Although spatial overlay analytics could help to find spatial relationships between different features, prior studies have identified some obvious problems in address matching when parcel data are used [20,21,22,23,24]. Even if the addresses of the parcels can be geocoded onto the street centerline to overlay with the geocoded physician data points, many of the physician addresses could still not be matched. Considering the spatial uncertainty within the geocoded results, matching two sets of uncertain data via spatial analytics could derive a certain portion of incorrect results.

The traditional procedure of geocoding has positional accuracy problems which has been studied in health services research [9,10,14,20,21,24,25,26]. Traditionally, street network data, such as Topologically Integrated Geographic Encoding and Referencing (TIGER) street centerline, have been used in the geocoding process to derive the location of a given address along with the road centerline through linear interpolation. Such a linear interpolation approach would generate high match rates, although the interpolated location may be largely biased from the real locations. This is especially significant in rural areas where more irregular street segments and fewer houses reduce the interpolation accuracy. In general, data quality of the input address table, errors in the reference data, and problems within the geocoding process would jointly contribute to the geocoding error.

Zandbergen introduced how to use parcel polygon or centroid as the reference data to implement a geocoding process in ArcGIS [22,23]. Since the input addresses are matched to parcel addresses, the geocoded locations are not necessarily aligned to street centerlines. Because the addresses assigned to the neighboring parcels are not necessarily continuous by street number or consistent by street names, the addresses in parcel data are discrete and non-linear information. Interpolation is not applicable in parcel-based geocoding process. As a result, it is reported that such an approach would produce much lower match rates, although it can achieve higher positional accuracy. This is partly due to the fact that parcel-based geocoding requires strict address matching as exact house numbers are taken into consideration when linear interpolation is not possible.

This paper introduces how to apply string match and text mining approaches to differentiate the home address from the practice address by comparing the string components within the addresses embedded in both physician data and parcel data. When addresses in different data sources could be formatted in different ways, developing string match and text mining solutions may not cover all heterogeneous scenarios completely. Visualizing the matching results could help identify and examine where and what the errors are and thus accumulatively help improve the solution. Eventually an optimized solution can be derived and extended to cover large datasets.

## 2. Study Area and Data

**Figure 1:**
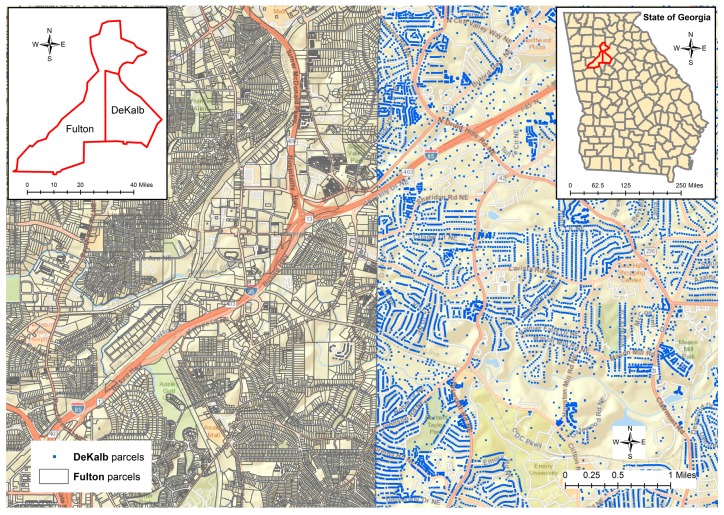
Sample parcel polygon data for Fulton County vs. parcel centroid data for DeKalb County.

As a pilot research project, two counties, Fulton and DeKalb, in the metropolitan area of Atlanta, Georgia were selected as the study area since both counties have parcel data available for this research. In the year 2014, Fulton County had 353,723 parcel polygons, while DeKalb County released 235,681 parcels as point features. Figure 1 displays the two different types of parcel data sets covering a part of the two counties, since the full scale of the parcel datasets cannot be clearly represented and differentiated in such a small figure. Both datasets contain the information about the unique identifier, address, and land use type of the parcel along with other information. The zoning code for the land use class of the parcel could reveal whether the parcels are used as agricultural, business, commercial, industrial, residential, exempted areas, or for other purposes. The class code in the parcel datasets helps to differentiate whether a certain address is a home address or not by checking whether the corresponding parcel is classified as a residential area or not.

The physician data used in this research is extracted from the 2014 the AMA Masterfile, which includes information about the physicians’ addresses, practice type, specialty, age, gender, employment settings, primary professions, medical school, Graduate Medical Education (GME) ending date, residency institutions, and other data. Within the document of Description of the AMA Physician Masterfile Data Elements [27], it is indicated that “A physician’s professional mailing address appears on the AMA Physician Profile. This address is typically assigned by the physician and can be either a home or office address.” Meanwhile, “A primary office address appears when it has been reported to the AMA.” Such ambiguous address information will certainly result in the problematic interpretation about the distribution of physicians, particularly when the location of the practice could hardly be differentiated correctly.

McLafferty et al. stated that “These data are problematic because a substantial proportion of physicians only report a mailing address, which is often the physician’s home (residential) location, rather than the address of the location where health care is provided.” [14]. In their research, both office and mailing addresses of the 2008 AMA Masterfile for the Chicago metropolitan region were geocoded by using the 2008 TIGER/Line files. In this way, the authors assessed the spatial mismatch between mailing and office locations in terms of the Euclidean distance. It was indicated that “almost half (47.6%) of physicians had a distance of zero indicating that the office and mailing addresses are identical”. However, there is no evidence and discussion in this paper about why and how a mailing address is often determined to be the physician’s home (residential) location.

A total of 6271 direct patient care physicians located within Fulton and DeKalb counties are further identified and extracted. Although the goal is to examine whether an address documented in the AMA Masterfile is a home address or not, a variety of uncertainty problems arose in the process of matching physicians’ addresses to the addresses in the parcel datasets. When the land use classification within the parcel dataset can be utilized to examine whether a given address is a residential location or not, matching the physicians’ addresses to the addresses of the parcels could be done by comparing the locations in the different datasets within a geographic information system (GIS). Since land parcel data are generally produced through a cadastral survey, which defines or develops the parcel boundary of the property as polygon features, conventionally the physicians’ addresses have to be first converted into geographic coordinates through the procedure of geocoding so that the location of the addresses can be presented as point features, to be integrated within a GIS for spatial analytics along with other datasets, such as parcel data.

## 3. Uncertainty in Data Processing and Spatial Analytics

This research is about how to match the street address information from two different sources. The address information in the parcel data is typically managed by a local government such as that of a county. In the case of physicians, their addresses were extracted from the AMA masterfile. Many of the physicians’ addresses cannot have an exact match in the parcel data. For this reason, geocoding and the direct application of spatial overlay may not be very useful. Consequently, we have to develop text mining solutions to directly process the string components in the street address. If there is no exact match, we can derive a fuzzy match. In this section, a variety of uncertainty problems are first introduced to establish the research context and background.

**Figure 2:**
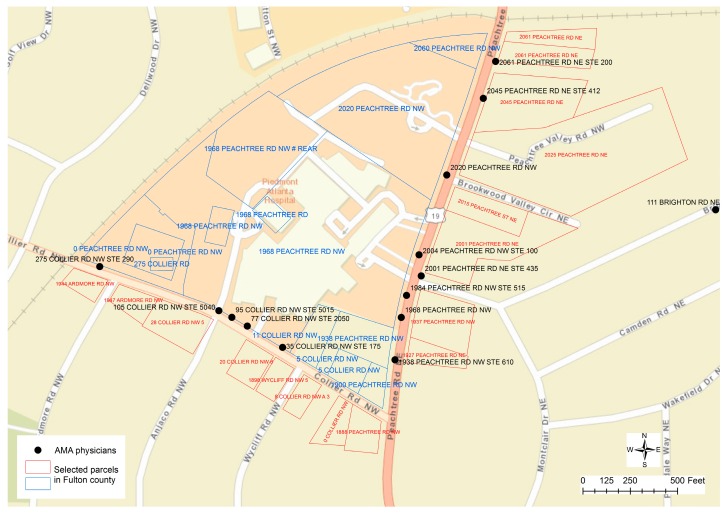
Discrete and inconsistent address assignment and distribution in a parcel dataset.

Figure 2 displays some typical scenarios about the addresses assigned to the parcels that could be beyond our normal cognition and comprehension about the distribution of street addresses along the roads. It can be found that many addresses assigned to the parcels along the street of “COLLIER RD NW” are actually using the street name of “PEACHTREE RD NW”. In fact, the street name for US Route 19 is “PEACHTREE RD NE”. The suffix of “NW” is only assigned to a few parcels on the west of US Route 19 that are used by the Piedmont Hospital. Particularly, the parcel of “275 COLLIER RD” is embedded within two other parcels that have the same street address of “0 PEACHTREE RD NW”, while the street numbers are not assigned continuously or consistently because this section of COLLIER RD only has two other street numbers of 5 and 11 then the street number jumped to 275. For the street addresses between “0 PEACHTREE RD NW” and “2060 PEACHTREE RD NW”, only four other street numbers are assigned to a few parcels as 1900, 1938, 1968, and 2020. The same street address can be assigned to multiple parcels. When address points, such as the centroid of parcel polygons, are used for geocoding, more problems could be involved as even the boundary of the property could not be perceived. For example, in Figure 1, when a large amount of spaces are distributed, the centroid of a parcel polygon cannot help to determine which address is closer to which parcel property.

**Figure 3:**
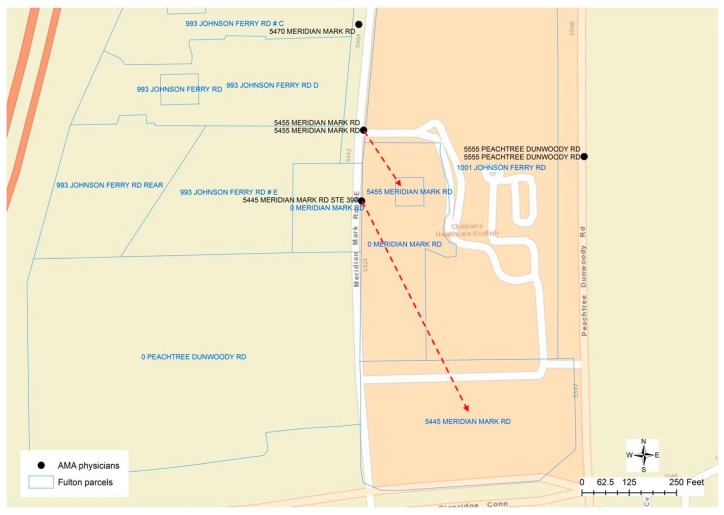
Physicians’ location vs. parcels that have the same address.

Although the result of geocoding would have inaccuracy problems, geocoded physician addresses could be helpful for researchers to perceive the spatial location and distribution of physicians from a macroscopic perspective. However, geocoding itself cannot help to differentiate whether the address provided by a physician is the home address or practice address. Matching the physicians’ addresses to the addresses in the parcel data through spatial overlay analytics could be problematic since the parcels nearest to the geocoded physician’s location may not be the correct parcels that have the same street address as the physicians. In the case of Fulton County that provides parcel data as polygon features, for example, the geocoded location of a physician may be far away from the corresponding parcel property. As described in Figure 3, several physicians are working at the same location “5445 MERIDIAN MARKS RD”. The parcels nearest to this geocoded point location have the address as “0 MERIDIAN MARKS RD”, while the corresponding parcel “5445 MERIDIAN MARKS RD” is 130 meters away displayed at the bottom of the Figure 3. Given the other physicians’ location at “5455 MERIDIAN MARKS RD”, the street address of the closest parcel is “1001 JOHNSON FERRY RD”, while the parcel that has the same address is about 45 meters away from the physicians’ geocoded location. When the parcel centroid data is used in DeKalb County, because the geocoded location is along the street centerlines, even if the physician has the same address as that in a parcel, there are two different point locations representing the centroid of a polygon and the approximate location along a street centerline. In this case, the nearest points from two different sources may not have the same street addresses.

While visualizing the geocoded physicians’ addresses along with the base map and parcel data can help understand the location and potential relationships between physicians’ location and the parcel polygons or centroids, matching physicians’ geocoded locations to the nearest parcels could hardly identify the correct connection between the two address sources. Alternatively, one possible approach is to geocode the addresses of the parcels in Fulton and DeKalb counties so that the addresses of both physicians and parcels are geocoded by the same street network data. In this way, if the address of a physician is exactly the same as the address in the parcel data, then they will be placed at the same geocoded location. For this reason, spatial overlay operation can be implemented to find the physicians whose addresses are the same as the addresses in the parcel data. Once such a connection can be established, the land use and zoning code of a parcel can then be used to clarify whether the physician’s address is a residential location or not.

Unfortunately, as the above discussion has revealed, the weakness and problem in using parcel data to derive geocoded locations is that a significant portion of physician data cannot be matched to the parcel data if the physician’s address is not exactly the same as the address in the parcel, because the linear interpolation approach is not applicable. Figure 2 displays a typical example to illustrate such a problem. Along the street “COLLIER RD”, five unique physicians’ locations can be found but only “275 COLLIER RD” can be matched to the address in the parcel data, while corresponding street addresses in the parcel data cannot be identified to match the other four addresses with street numbers of 35, 77, 95, and 105. Similarly, two other physicians’ addresses, “1984 PEACHTREE RD NW” and “2004 PEACHTREE RD NW”, cannot be matched to any address in the parcel data. In conclusion, spatial overlay analytics could only partially resolve the problems to clarify the spatial uncertainty problem.

## 4. Differentiating Home Addresses from Practice Addresses through Text Mining

Considering the parcel data have high positional accuracy and have the necessary information to differentiate whether an address is a home address or not, appropriate solutions have to be developed to find the parcels that are nearest to a given physician’s address, in case the physician’s address does not have an exact match to any address in the parcel data. Since linear interpolation is not applicable to derive the location when parcel data is utilized, the components in each physician’s address have to be compared to the addresses in the parcel data. Such a challenge can be resolved by developing solutions of string comparison and text mining.

Text mining is one approach in data mining that processes unstructured text data, which is composed of strings. Text mining helps to understand data pattern embedded in the texts and to extract appropriate information for knowledge discovery from a text document [28,29]. In this pilot study, the address information in both physician and parcel data is captured and stored as strings. The common workflow and algorithm for string processing and text mining can be applied to provide a flexible and controllable solution to do address matching between different datasets. In this study, an intuitive three-step workflow is developed to clean the address strings, tokenize the string components, and match the addresses between physician and parcel data.

When the physicians complete the survey, they may not have any standard to follow to submit the address information, which is most probably in a personalized format. Examining and cleaning the input address will help to transform the address information in a more uniform format for further manipulation. This procedure may include multiple tasks, such as the removal of extra punctuation marks, changing all characters to lower case, before other basic string operations are implemented. As common practice, two input items would be provided in the survey to allow the users to provide address information. Correspondingly, for each physician, two attribute columns are used to record the address information. While most of the physicians provide the street address information in the first column, some physicians put the name of the hospital or organization in the first column and the street address in the second column. For this reason, the street address has to be identified and extracted or merged into a single column.

Tokenization is the process of breaking a sequence of words into semantically meaningful segments. Ideally, different components of an address are recorded separately or written in one field, but strictly conform to a universal format (e.g., U.S. Postal Service standard address format). While the parcel datasets are well tokenized, the physicians’ addresses have to be decomposed and normalized in the same format and style as that of the parcel data, so as to be compared with the parcel datasets.

Wong & Chuah proposed a hybrid approach to normalizing the address by different strategies [30]. One approach enumerates all possible combinations of address components (e.g., zip code, street name, city name) and compares each of them with the reference addresses. This approach becomes problematic as the reference database contains a large number of features. Particularly when millions of parcel data are used, such an approach may not be efficient and effective. Alternatively, a set of rules can be used to extract address components based on keywords and word positions. For example, directional terms are commonly written as “N”, ”S”, ”W”, ”E”, and their pairwise combinations can be identified as keywords in an address. Street suffixes and types can also be enumerated in a small dictionary, allowing for direct identification in an address. This approach relies on well-designed rules but could be too sophisticated to update.

In this pilot study, the rule-based address tokenization and address matching algorithms are applied to compare the physician’s address to the addresses in the parcel datasets. Since parcel data contains a large volume of records, a spatial limitation can be applied to reduce the search query by specifying a buffer distance around the geocoded physician’s location. In most cases, the geocoded locations are positioned correctly onto the street network system. In a few rare cases, however, if the geocoded locations are not correct, the spatial limitation may not help to find the correct parcel record accordingly. Without using a spatial buffer constraint to narrow down the scope of the search query over a large volume of parcel datasets, we applied a hierarchical search to minimize the computational load. First of all, the unique zip code in the physicians’ addresses has to be identified and extracted. Physicians are then grouped by zip code, which is further used to retrieve a subset of parcel data with the same zip code for comparison.

When comparing the string of address components of a physician to the address of parcels, unless they are exactly the same, approximate string matching solutions have to be explored and applied to find the potential match. In this study, Levenshtein distance is used to measure the similarity between two strings. Levenshtein distance calculates the least number of necessary single-character operations to transform a string to another. Allowed operations include insertion, deletion and substitution [31,32,33]. For example, string “FLAT SHOULS” needs one substitution operation to match the string of “FLAT SHOALS”. In this case, the Levenshtein distance is one. As a result, two strings are considered as equivalent when the number of operations needed to make them exactly the same is under a certain threshold number. This approach is employed in our study to solve the misspelling problem in street names among different data sources.

Once all physicians’ addresses are well-formatted in the complete or standard form as “street number + prefix direction (optional) + street name + street type or suffix + post direction (optional)”, the following workflow and rules are applied to process and match the street addresses between the physician data and parcel data through multiple steps.

1. Look through the physician dataset to clean the address. If a single character is attached to the street number, only the number will be maintained, while all punctuation is removed (e.g., change “1000B Main St.” or “1000-B Main St.” to “1000 Main St”).
2. Choose a naming convention to define certain types of street names. For example, numbers in street names are transformed to their alphabetical formats (e.g., 1st AVE to FIRST AVE). In the case of local landmarks that are common sense in a region, abbreviations would be used in different styles. For example, “MARTIN LUTHER KING JR” could be recorded as “MLK”, “M L K”, “M.L.K.”, “M. L. K.”, or “ML KING”, while “JR” could be included in some of the addresses but not all of them. In this case, a uniform name has to be used to replace different styles.
3. Tokenize physicians’ addresses into string components. Regular expression and a series of procedures are used to examine and transform the input of physicians’ addresses into the complete or standard form as mentioned above.
4. Compare the string components of the physician’s address with the corresponding address components in the parcel data. By applying the spatial limitation or the filter of a zip code to retrieve partial parcel datasets, the physician’s address is compared to each address of the selected parcel addresses through the following procedures:
  (a) Check if the physician’s address is empty or invalid, such as if the address does not start with a street number, or a PO Box is used.
  (b) Compare the parcel’s street name with the physician’s street name. Levenshtein distance is calculated between the two street names to examine potential misspelling in the street name. If Levenshtein distance is 0, it is a perfect match. If Levenshtein distance is 1, it means one character is different between the two address names. This could probably be a misspelling error, and thus the two addresses could be matched.
  (c) Compare parcel’s street suffix with the physician’s street suffix to see if they are the same or not.
  (d) Compare the parcel’s directional terms to the directional terms in the physician’s address. The comparison of directional terms is only conducted when both physician and parcel have the directional terms.
  (e) Compare the parcel’s street number to the street number in the physician’s address. If they are not exactly the same, the parcel with the street number nearest to the physician’s street number will be selected as the match address to the physician’s address. In this case, whether the street number is an even or odd number will be critical. If the physician’s street number is an even number, then only parcels with even street numbers will be considered, otherwise the parcel would be on the opposite side of the street and could not be matched to the physician’s address. For example, Figure 2 displays four unique locations on the street “COLLIER RD NW” for physicians whose street numbers (35, 77, 95, and 105) cannot find an exact match to the nearby parcels (5, 11, 275, 8, 18, 20, 28). Parcels with even street numbers are on the other side of the hospital and are residential areas. If the physicians’ addresses are matched to the parcels with closest street numbers, these physicians’ addresses could be classified as home addresses, meaning that the result is wrong. Differentiating odd and even street numbers should be critical in deriving the correct conclusions.
  (f) In the case that one physician’s address would have multiple matches to the addresses in the parcel data, the selection procedure could be a little bit more complicated. If all parcels have the same land use code, it is easy to clarify the physician’s address as the same land use type. However, if these parcels share a different land use classification code, then the land use type of the corresponding physician’s address could hardly be clarified. For example, in response to one physician’s address “199 14TH ST NE”, there are 89 parcels that have the same address “199 FOURTEENTH ST” in Fulton County’s parcel data. Four of them have the land use code as “C1” (i.e., Commercial) and 85 of them have the code as “R3” (i.e., Residential). In this case, even if the address can be matched exactly, the land use type cannot be determined without knowing the concrete assignment of the parcel polygon to the physician’s address.
5. The unmatched cases have to be manually or visually examined to understand the potential problems in the address matching procedures for further improvements.

The result of the address matching is summarized in Table 1. Among 6271 physicians in Fulton County and DeKalb County, a small amount of physicians (about 8%) did not provide any street address information or their street addresses are not valid. Within the remaining 92% of physicians, about 4% of the street addresses cannot find corresponding counterparts in the parcel datasets. While 88% of physicians’ addresses could be matched to parcel addresses, 81% can be identified as practice addresses, 6% as home addresses, and 1% could not be determined when multiple parcels share the same street address but have different land use codes. Within the total number of matched addresses, 30% of the home addresses (i.e., 121) have an exact match with the addresses in the parcel data, 57% of the practice addresses (i.e., 2907) have an exact match with the addresses in the parcel data. It can be concluded that address matching through string comparison and text mining could be the applicable and efficient solution to differentiate whether a given address is a residential location or not, so that such a spatial uncertainty problem can be reduced or mitigated. In this pilot study, an office address in the 2014 AMA Masterfile is processed. The result is significantly different from the prior work [14], which has a different time frame and study area.

In prior works, the geocoding process might try to maximize the opportunity to derive a location, or fuzzy location. For example, if no street address is provided, it may use the centroid of city, zip code, or state polygon as the location for this record. In this study, when text mining is applied to process two datasets, if one record in physician data has no street address, it cannot be matched to anything in the parcel datasets. There is no maximization process in this study.

## 5. Results and Discussion: Visual Examination and Validation

Although computer aided automation approaches could be explored and developed to locate the addresses of the physicians onto maps, and to compare the addresses to the information captured in the parcel data to determine whether the address is a residential location or not, such computer programs cannot tell whether the result of geocoding or address matching is correct or not. For this reason, a visual reasoning approach has to be developed to analyze and examine the results derived by the software or computational modules. In fact, those rules and workflow discussed above have been improved and updated through iterative and retrospective visual analytics that help to advance our cognition and apprehension of the errors or problems in the processes of geocoding and address matching.

**Figure 4:**
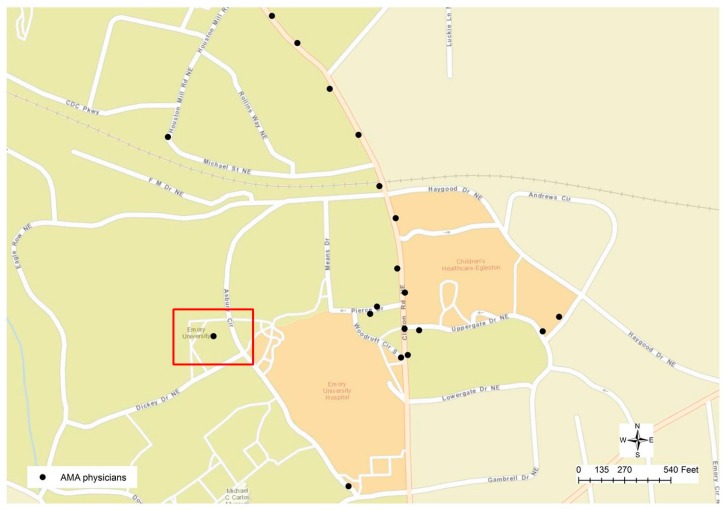
Errors in the geocoding results.

Figure 4 displays a typical geocoding error that was ignored until it could be identified through visual examination. After some initial operations in address matching were performed, it was noticed that some physicians’ addresses could not be matched to any surrounding parcels. The highlighted point in Figure 4 is actually a cluster of 161 physicians whose addresses are geocoded to the same location. For this reason, when we zoom to the targeted area on the map, it can be found that this cluster of physicians is close to Emory University Hospital. By examining the addresses in the attribute table, a combination of problems can be found since some of the physicians’ addresses are empty or not valid, while some of them do have the valid addresses but they were geocoded to the wrong location due to the maximization of location matching in the geocoding process. By examining this point location to the other points on the map in Figure 4, it can be found that all other geocoded locations are along the road centerlines, while this problematic location is not close to any road. For this reason, visual reasoning helps to derive a conclusion as a geocoded location could be wrong if it is not along the street centerline. In general, a hybrid method was applied in the geocoding process to increase matches. For example, in case that the address information in the physician profile was not complete, when the zip codes were available in the profile, then such zip codes from the preferred mailing addresses were utilized to accomplish the geocoding process. Such locations would most likely fail to be geocoded at street level. Particularly since the text mining and string matching approach could generate a matching result to a higher degree, we can find the differences in the results derived from two different approaches.

**Figure 5:**
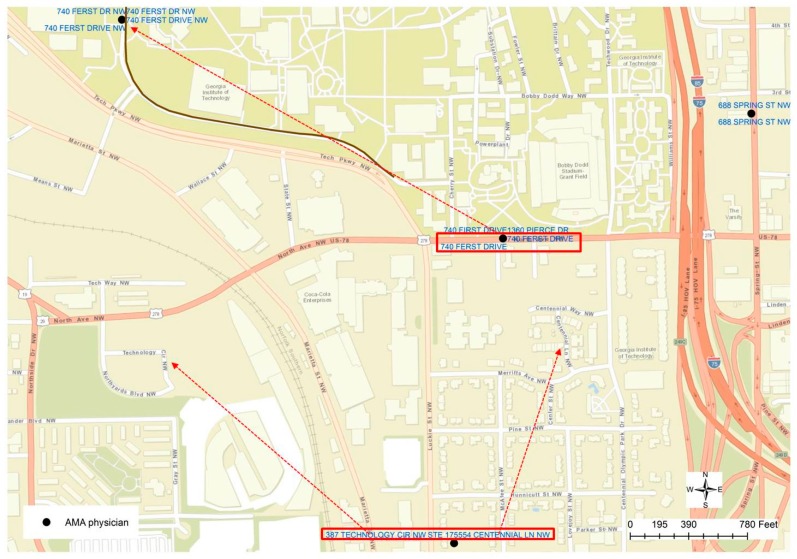
More errors in the geocoding results.

Such a conclusion could help to find other errors generated from the geocoding process, such as the other case displayed in Figure 5. The highlighted point location is not positioned along the road centerline, but is within a community block. By checking the addresses in the attributed table, it could be found that two different addresses are recorded in the table, which has proved to be another mistake in the geocoded result. By visually examining the nearby address location, one other error could be identified since two different addresses are labeled at the same location, although the geocoded location is along a road or in the street centerline. Through a further examination, it can be found that those two addresses (“740 FERST DR” and “1360 PIERCE DR”) are geocoded at the wrong location. “740 FERST DR” is located at the upper left corner of Figure 5, while actually “PIERCE DR” can be found in Figure 4, highlighted in the blue rectangle. As a result, a new rule can be derived as if a cluster of the geocoded location is positioned at the same x and y coordinates but the street addresses are not the same, such a cluster of physicians’ addresses may contain errors. Such a rule, however, is not valid to the parcel dataset, in which duplicated parcel polygons may have exactly the same geometric boundary or location but have different addresses. By applying such rules, it can be found that each of the 13 unique locations has multiple addresses, indicating potential geocoding errors.

Besides discovering potential mistakes in the geocoding results, the visual reasoning approach is critical to helping to understand the variety of problems in matching the addresses between two different datasets that were beyond the automation procedures and our common cognition. Obviously, a computer is not “intelligent” enough to find the errors, thus visualizing the matching results did indeed help us to reason, for example, why several clusters of physicians were assigned to a home address or an uncertain match, though visually, they were distributed near hospital areas. For this reason, we have to visually examine the datasets and matching results on the map to identify and understand the causes of the errors, so as to make further improvement on the methods. Many useful cases were identified by visual examination, therefore we understood what is wrong in the matching procedures so that we could improve the rules applied in the address matching workflow.

As a typical scenario, if any cluster of physicians’ addresses are classified as residential locations, or cannot be matched to the nearby parcel data, it would imply a potential problem. For example, in Figure 2, several clusters of physicians’ locations on the street “COLLIER RD NW” were initially classified as residential locations. It will be strange if 77 physicians’ homes are located at the same address as “35 COLLIER RD NW”. Such an error was generated in the fuzzy matching process without any consideration of odd or even street numbers. In this case, the physicians’ addresses (35, 77, 95, and 105) could be matched to the parcels on the other side of the road with even street numbers (8, 18, 20, 28) if odd street numbers (5, 11, 275) are ignored.

**Figure 6:**
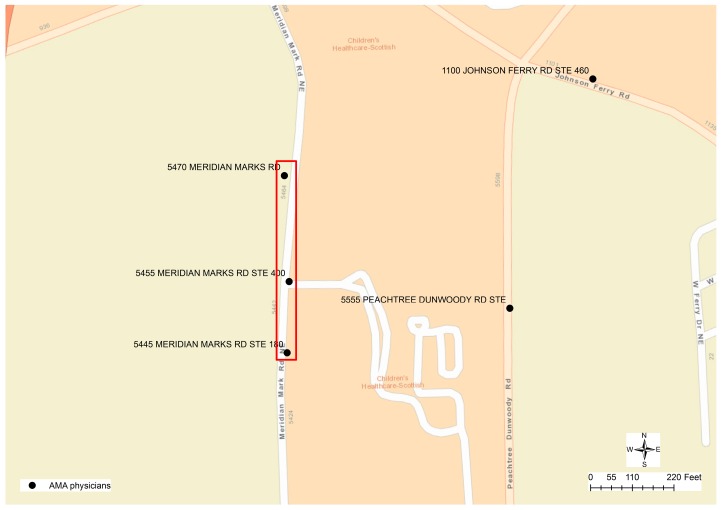
Misspelling error in physician data.

**Figure 7:**
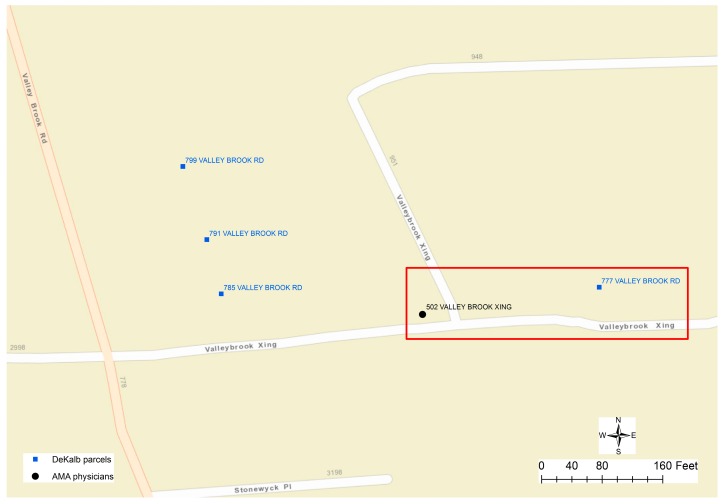
Misspelling error in parcel data.

In another example, Figure 6 displays three clusters of 50 physicians’ addresses that cannot be matched to nearby parcel addresses. Eventually, the difference in the street name was identified as the cause of this problem. The street name of physicians’ addresses is recorded as “MERIDIAN MARKS RD”, while in the parcel data, the street name is “MERIDIAN MARK RD”. While the parcel datasets are used as the reference data to compare and match addresses in physicians’ data, regrettably, the parcel datasets also contain potential errors. Figure 7 displays a mismatch, in which the physician’s address is “502 VALLEYBROOK XING”, while the only address for parcels along “VALLEYBROOK XING” could be wrongly documented as “777 VALLEY BROOK RD”. As a conclusion, errors in the addresses of both physicians’ and parcel datasets could result in unmatched cases.

## 6. Conclusions

Combined approaches of spatial analytics and text mining with visual examination are the feasible and efficient ways to clarify and identify the uncertainty in physician practice location. Although the address matching procedures could differentiate, regarding whether a given address is a residential location or not, the quality of physicians’ addresses and the addresses in the parcel data will have a significant impact on the matching result. Since both datasets contain error messages, unmatched addresses are inevitably expected. Through this study, the potential errors in the geocoded locations can be identified by examining whether the location is along a street centerline, or whether the same location has multiple different addresses.

When developing rules for string match and text mining over address information from heterogeneous sources, visual reasoning and examination should be the necessary process that helps to understand the cause of the errors and to improve the rule development. Particularly for local landmarks and well-known conventions, abbreviated terms could be used in varied formats that increase the difficulty in address matching. However, one distinguishing characteristic in physicians’ addresses could be a critical indicator for visual reasoning and examination in the case of a cluster of physicians’ addresses being matched to one residential location, because it is unrealistic that so many physicians live at the same address, or cannot be matched to anywhere, although many physicians could, for example, work in the same building of a hospital.

## Figures and Tables

**Table 1:** The result of address string matching between physician and parcel datasets.

Type of Address via the Matching Process		Number of Addresses	Percentage
Empty addresses		488	7.78%
Invalid addresses		18	0.29%
Matched addresses	residential addresses	401 (121)	6.39%
	non-residential addresses	5063 (2907)	80.74%
	Undetermined	50	0.80%
Unmatched addresses		251	4.00%
Total		6271	100%

## References

[1] Council on Graduate Medical Education (COGME) (1998). Physician Distribution and Health Care Challenges in Rural and Inner-City Areas: Council on Graduate Medical Education Tenth Report.

[2] Council on Graduate Medical Education (COGME) (2010). Advancing Primary Care: Council on Graduate Medical Education Twentieth Report.

[3] Ricketts T.C., Goldsmith L.J., Randolph R., Lee R., Taylor D.H., Ostermann J. (2007). Designating places and populations as medically underserved: A proposal for a new approach. J. Health Care Poor Unders..

[4] Xierali I., Bazemore A.W., Phillips R.L., Petterson S.M., Dodoo M.S., Teevan B. (2008). A perfect storm: Changes impacting Medicare threaten primary care access in underserved areas. Am. Fam. Phys..

[5] Zhang X., Phillips R.L., Bazemore A.W., Dodoo M.S., Petterson S.M., Xierali I., Green L.A. (2008). Physician distribution and access: Workforce priorities. Am. Fam. Phys..

[6] Peterson L.E., Bazemore A.W., Bragg E.J., Xierali I.M., Warshaw G. (2011). Rural urban distribution of the US geriatrics physician workforce. J. Am. Geriatr. Soc..

[7] Health Resources and Services Administration Lists of Designated Primary Medical Care, Mental Health, and Dental Health Professional Shortage Areas. https://www.federalregister.gov/documents/2016/07/01/2016-15678/lists-of-designated-primary-medical-care-mental-health-and-dental-health-professional-shortage-areas.

[8] The Patient Protection and Affordable Care Act. https://www.gpo.gov/fdsys/pkg/BILLS-111hr3590enr/pdf/BILLS-111hr3590enr.pdf.

[9] Bonner M.R., Han D., Nie J., Rogerson P., Vena J.E., Freudenheim J.L. (2003). Positional accuracy of geocoded addresses in epidemiologic research. Epidemiology.

[10] Wu J., Funk T.H., Lurmann F.W., Winer A.M. (2005). Improving spatial accuracy of roadway networks and geocoded addresses. Trans. GIS.

[11] Bichler G., Balchak S. (2007). Address matching bias: Ignorance is not bliss. Polic. Int. J. Police Strateg. Manag..

[12] Drummond W.J. (1995). Address matching: GIS technology for mapping human activity patterns. J. Am. Plan. Assoc..

[13] Goldberg D.W., Wilson J.P., Knoblock C.A. (2007). From text to geographic coordinates: The current state of geocoding. URISA J..

[14] McLafferty S., Freeman V.L., Barrett R.E., Luo L., Shockley A. (2012). Spatial error in geocoding physician location data from the AMA Physician Masterfile: Implications for spatial accessibility analysis. Spat. Spat. Temporal Epidemiol..

[15] Hay G., Kypri K., Whigham P., Langley J. (2009). Potential biases due to geocoding error in spatial analyses of official data. Health Place.

[16] Mazumdar S., Konings P., Butler D., McRae I.S. (2013). General practitioner (family physician) workforce in Australia: Comparing geographic data from surveys, a mailing list and medicare. BMC Health Serv. Res..

[17] Bell S., Wilson K., Shah T.I., Gersher S., Elliott T. (2012). Investigating impacts of positional error on potential health care accessibility. Spat. Spat. Temporal Epidemiol..

[18] Alma J., Buschmanb R.D., Sjoquistb D.L. (2011). Rethinking local government reliance on the property tax. Reg. Sci. Urban Econ..

[19] Geoghegan J. (2002). The value of open spaces in residential land use. Land Use Policy.

[20] Cayo M.R., Talbot T.O. (2003). Positional error in automated geocoding of residential addresses. Int. J. Health Geogr..

[21] Zimmerman D.L., Fang X., Mazumdar S., Rushton G. (2007). Modeling the probability distribution of positional errors incurred by residential address geocoding. Int. J. Health Geogr..

[22] Zandbergen P.A. (2008). A comparison of address point, parcel and street geocoding techniques. Comput. Environ. Urban Syst..

[23] Zandbergen P.A. (2009). Geocoding quality and implications for spatial analysis. Geogr. Compass.

[24] Jacquez G.M. (2012). A research agenda: Does geocoding positional error matter in health GIS studies?. Spat. Spat. Temporal Epidemiol..

[25] Goldberg D.W., Jacquez G.M., Mullan N., Boscoe F. (2013). Geocoding and health. Geographic Health Data: Fundamental Techniques for Analysis.

[26] Rushton G., Armstrong M.P., Gittler J., Greene B.R., Pavlik C.E., West M.M., Zimmerman D.L. (2006). Geocoding in Cancer Research: A Review. Am. J. Prev. Med..

[27] American Medical Association Description of AMA Physician Masterfile Data Elements. http://www.ama-assn.org/ama1/pub/upload/mm/eProfiles/mm/mfile_elements.pdf.

[28] Hotho A., Nürnberger A., Paaß G. (2005). A Brief Survey of Text Mining. LDV Forum.

[29] Gupta V., Lehal G.S. (2009). A survey of text mining techniques and applications. J. Emerg. Technol. Web Intell..

[30] Wong W.S., Chuah M.C. (1994). A hybrid approach to address normalization. IEEE Expert.

[31] Levenshtein V.I. (1966). Binary codes capable of correcting deletions, insertions, and reversals. Sov. Phys. Dokl..

[32] Hall P.A., Dowling G.R. (1980). Approximate string matching. ACM Comput. Surv..

[33] Ukkonen E. (1985). Algorithms for approximate string matching. Inf. Control.

